# SMORE: Synteny Modulator of Repetitive Elements

**DOI:** 10.3390/life7040042

**Published:** 2017-10-31

**Authors:** Sarah J. Berkemer, Anne Hoffmann, Cameron R. A. Murray, Peter F. Stadler

**Affiliations:** 1 Bioinformatics Group, Department of Computer Science, and Interdisciplinary Center for Bioinformatics, University of Leipzig, Härtelstraße 16-18, D-04107 Leipzig, Germany; bsarah@bioinf.uni-leipzig.de (S.J.B.); anneh@bioinf.uni-leipzig.de (A.H.); 2 Max Planck Institute for Mathematics in the Sciences, Inselstraße 22, D-04103 Leipzig, Germany; 3 Department of Biochemistry, University of Alberta, Edmonton, AB T6G 2H7, Canada; crmurray@ualberta.ca; 4 German Centre for Integrative Biodiversity Research (iDiv) Halle-Jena-Leipzig, Competence Center for Scalable Data Services and Solutions, and Leipzig Research Center for Civilization Diseases, University Leipzig, D-04103 Leipzig, Germany; 5 Fraunhofer Institute for Cell Therapy and Immunology, Perlickstrasse 1, D-04103 Leipzig, Germany; 6 Institute for Theoretical Chemistry, University of Vienna, Währingerstraße 17, A-1090 Wien, Austria; 7 Center for RNA in Technology and Health, Univ. Copenhagen, Grønnegårdsvej 3, DK-1870 Frederiksberg C, Denmark; 8 Santa Fe Institute, 1399 Hyde Park Rd., Santa Fe, NM 87501, USA

**Keywords:** bioinformatics, pipeline, workflow, concerted evolution, synteny, orthology, tandem duplications, Y RNAs, tRNAs

## Abstract

Several families of multicopy genes, such as transfer ribonucleic acids (tRNAs) and ribosomal RNAs (rRNAs), are subject to concerted evolution, an effect that keeps sequences of paralogous genes effectively identical. Under these circumstances, it is impossible to distinguish orthologs from paralogs on the basis of sequence similarity alone. Synteny, the preservation of relative genomic locations, however, also remains informative for the disambiguation of evolutionary relationships in this situation. In this contribution, we describe an automatic pipeline for the evolutionary analysis of such cases that use genome-wide alignments as a starting point to assign orthology relationships determined by synteny. The evolution of tRNAs in primates as well as the history of the Y RNA family in vertebrates and nematodes are used to showcase the method. The pipeline is freely available.

## 1. Introduction

A precise record of the history of a gene family, that is, an accurate reconstruction of a phylogenetic gene tree, is an indispensable prerequisite for a detailed description of the functional evolution of its members and the assessment of innovations [1,2]. The exact placement of gene duplication and gene-loss events relative to a species tree is also of key importance in the context of forward genomics [3]. The first crucial step towards elucidating the history of a gene family is to distinguish orthologs, that is, gene pairs that originated from a speciation event, from paralogs, which arose by gene duplication [4]. A large arsenal of computational methods has become available to determine orthology. These tools either compute a gene phylogeny from aligned sequences and subsequently reconcile the gene tree with a species tree; otherwise they use a “reciprocal best match” rule [5,6]. We refer to [7,8,9,10,11] for reviews of the topic and benchmarks of the most commonly used tools. Both approaches assume that genes evolve essentially independently so that sequence divergence is a faithful measure of evolutionary distance.

Multicopy genes sometimes violate this assumption in a very strong way. Concerted evolution [12,13] may cause paralogous genes to maintain essentially identical sequences over long evolutionary time scales. The underlying mechanism is primarily homologous recombination, which leads to gene conversion, in which, a piece of the sequence from one copy of the gene effectively overwrites a homologous region in another copy. Unequal crossover between repeating units and gene amplification are also important contributors (e.g., [14]). Gene conversion is responsible for preventing the divergence of the individual copies of transfer ribonucleic acids (tRNAs) [15], small nuclear RNAs (snRNAs) [14], the ribosomal RNA (rRNA) cistron [16], and the histone genes [17]. Paralogous genes can escape from concerted evolution [18] and then rapidly accumulate mutations typically leading to a loss of function and hence eradication from the genomic record. Together, these processes can result in a rapid net turn-over of gene copies and sometimes large differences in the number of copies in closely related genomes. This effect has been studied in much detail, in particular for the case of tRNAs [19,20,21,22,23].

Because paralogous sequences are essentially identical, it is not possible to identify orthologs of genetic elements that are subject to concerted evolution by means of sequence comparison. Synteny, however, provides a potentially powerful means of discriminating between orthologous loci. Reliable information of synteny can be obtained whenever there are unique sequence regions in close genomic proximity to the locus of interest. Here, orthology can be established with high confidence among related species. The conservation of proximity to such independently evolving regions can then be used to distinguish orthologous from paralogous copies of the ambiguous sequence element. This idea has been exploited in the past, in particular as a means of tracing the evolution of tRNAs [19,20,21,22]. In [23], we explored its implication in some detail and proposed a more systematic conceptual workflow for the evolutionary analysis of multicopy genes that can use genome-wide multiple sequence alignments (MSAs), many of which are already publicly available, as a source of synteny information. In the present contribution, we describe an implementation of a fully automatic computational pipeline that serves as a convenient tool for this purpose, and we describe applications to two classes of non-coding RNAs (ncRNAs).

The origin of tRNAs was from before the separation of the three domains of life. There is clear evidence, furthermore, that all tRNA genes are homologs, derived from an ancestral “proto-tRNA” [24], which in turn may have emerged from even smaller components [25]. These are indispensable in all organisms. In addition to their ancestral role as mediators of the genetic code (e.g., [26]), tRNAs have secondarily acquired additional functions, reviewed, for example, in [27,28]. Beyond bona fide tRNAs, there is a rich universe of tRNA-derived repetitive short interspersed nuclear elements (SINEs) [29] and small RNAs that either directly derive from tRNAs [30,31] or arose indirectly as exapted SINEs [32]. Multiple identical copies, often large numbers of pseudogenes, and rapid, lineage-specific expansions of particular families are typical for tRNA evolution, at least in Eukarya [19,33]. Among the elements under concerted evolution, tRNA genes are the most widely studied elements. They show a rapid turnover as the consequence of frequent seeding of new loci compensated for by high rates of pseudogenization [19,20,21,22]. While gain and loss events can be estimated from changes in the total number of paralogs with often acceptable precision for low-copy-number gene families such as microRNAs [34], this is not the case for tRNAs, as the number of conserved tRNA loci very quickly decreases with phylogenetic distance [19,23].

The second example are mammalian Y RNAs. Like tRNAs, Y RNAs are pol III transcripts [35]. They form the RNA component of Ro ribonucleoprotein (RoRNP) particles [36,37]. The molecules exhibit a characteristic secondary structure that has been extensively studied in the past [38,39]. They are essential for the initiation of chromosomal deoxyribonucleic acid (DNA) replication in vertebrates [40], likely in conjunction with the origin recognition complex [41]. As part of the RoRNP, they are involved in RNA stability and cellular responses to stress [42]. In addition, small RNA fragments are enriched in apoptotic cells [43]. The evolution of Y RNAs has been studied in some detail in [44], indicating a single, evolutionary conserved genomic cluster comprising four paralog groups designated Y1, Y3, Y4, and Y5. With the notable exception of mammals, which harbor on the order of 1000 Y RNA-derived retro-pseudogene sequences [45], most other vertebrates show only a few Y RNA-derived pseudogenes.

## 2. Methods

### 2.1. Overview

**Figure 1:**
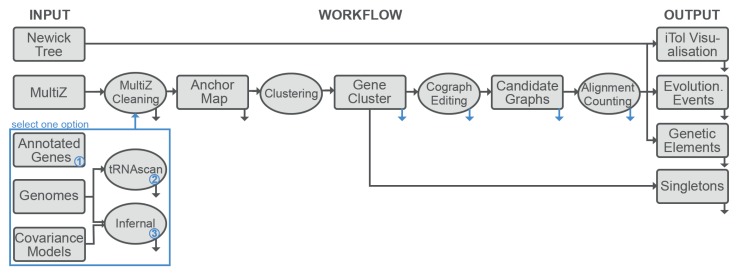
Summary of the computational workflow implemented in the Synteny Modulator Of Repetitive Elements (SMORE) pipeline for analyzing the evolution of mutlicopy genes. The compilation of orthology estimates and the quantitative analysis are logically separated and can also be used independently of each other; see text for details. The blue box describes options for input data. Black arrows pointing toward the next step of the pipeline (to the right) show an uninterrupted workflow and hence no printing or reading of files in between single steps of the pipeline. Black arrows pointing downward indicate output files that are always part of the output, whereas blue arrows pointing downward indicate the creation of temporary files and of optional output for the user.

The pipeline is composed of two modular parts: (i) the inference of the orthology relation, and (ii) the quantitative analysis of the orthology relation (see Figure 1). The first component identifies a map of genomic anchor points that are used to partition the annotated elements of interest into an initial set of candidate clusters. These are then processed to account for the most common artefacts in the input data and are refined using information that is provided by analyzing related but distinguishable sequence elements together. The second part of the pipeline is largely independent of the first and can also be employed using input data generated by other, third-party methods. With our pipeline, we provide an uninterrupted workflow that returns results based on input files and user-defined parameters. With the exception of breaks between subcommands indicated in Figure 1 and where output data is provided for the user, UNIX pipes are utilized to transfer data between software components.

### 2.2. Annotation of the Loci of Interest

In this contribution, we discuss two showcase examples. In each case, the first step is the identification of the loci of interest. Different tools and initial data have been used. We employed tRNAscan-SE [46] to annotate nuclear tRNA genes in up to 10 mammalian genomes. We identified Y RNA genes starting from the Y RNA sequences reported in [44] for mammals and a sequence alignment in [47] for nematode genomes. For the mammalian sequences, we first constructed a MSA together with a consensus secondary structure using mlocarna [48,49,50]. To this end, we used Infernal [51] to generate and calibrate covariance models, on the basis of both multiple alignments. In the final step, Infernal was used to identify significant matches in the genome. The alignment of Y RNA sequences and information on the investigated genomes can be found in the Supplementary Materials S1 and S2, respectively.

### 2.3. Genomic Anchors

A key step in our workflow is the identification of *genomic anchors*. Following [23], we define a genomic anchor as a sequence interval for which orthology between pairs of genomes can be established without ambiguity. As it is key to our approach, we briefly review the concept here in more formal terms:

Given a genetic element $g_{A}$ of interest in species *A*, we make two assumptions:

1. For the genetic element $g_{A}$, we can find two flanking regions $p_{A}$ and $q_{A}$ that have orthologous counterparts $p_{B}$ and $q_{B}$ in species *B* on the basis of sequence similarity.
2. On the basis of genomic coordinates, the order of the sequences is determined such that $p_{A}<g_{A}<q_{A}$ and $p_{B}<q_{B}$.

As orthologous counterparts of genomic anchors might not be present in all species of interest, we define tight anchors. Here, we take the closest possible anchors for a given element $g_{A}$ in species *A* despite that there are no orthologous anchors in any other species. This ensures that the definition of orthologous genomic regions is as highly resolved as possible in the first step. The nature of genomic anchors is irrelevant and can be any sequence block.

Our starting point for the computation of genomic anchors is a MSA. We emphasize that MSAs in general do not correctly align multicopy genes, as well-conserved multicopy elements are often used for the generation of anchors for the MSA itself. This creates artefacts, because the initial alignment step by construction cannot distinguish between the individual copies of a family of loci that is subject to concerted evolution. We refer to [23,52] for a more extensive discussion of this issue. For mammals, we used the MultiZ alignment of 19 mammalian genomes with humans [53] and for nematodes, and the MultiZ alignment of 25 nematode genomes with *Caenorhabditis elegans* [54], downloadable through the University of California Santa Cruz (UCSC) Genome Browser. As a result of the duplicated genome regions and the presence of other multicopy elements, not all alignment blocks reported in the initial MultiZ alignments can meaningfully serve as genomic anchors. We therefore eliminated all genomic anchors, also called multiple alignment format (MAF) blocks, that overlapped with any element of interest or other MAF blocks of the MSAs.

In the final step, the MAF blocks immediately upstream and downstream of each annotated occurrence of an element of interest are compiled. Together, they form the *anchor map* for the family of genetic elements in question.

### 2.4. Candidate Clusters of Co-Orthologous Genes

**Figure 2:**
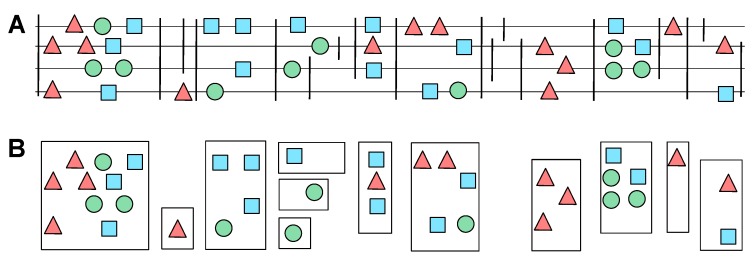
Example of syntenic gene clusters before post-processing. Panel (**A**) shows the initial genomic organization of different genetic elements (colored circles, triangles and squares). Grey horizontal lines represent genomes of different species. Vertical lines denote the genomic anchors, which in our setting correspond to unique multiple alignment format (MAF) blocks. These anchors subdivide the genetic elements into the syntenic gene clusters shown in panel (**B**). Genetic elements belonging to the same cluster are surrounded by a box.

The anchor map partitions the set of genetic elements into groups of potential co-orthologs. More precisely, we make the simplifying assumption that no genomic arrangement has occurred between the tight anchors enclosing an element. An initial set of clusters is obtained by combining only sets of elements that share the same pair of anchors. As shown in Figure 2, this may lead to (i) clusters that contain multiple elements from the same species, and (ii) the separation of elements into different clusters because of a lack of common anchors. The first case likely identifies in-paralogs, that is, recent duplications in one species. The second case may arise from deletions of the anchor elements in some species. More likely, however, it is associated with missing data or assembly artefacts. In the initial partition, this often produces a large number of singletons, which would lead to a substantial underprediction of orthology. To account for these issues, we post-process the initial clusters.

In order to deal with missing anchors, we join clusters $C^{\prime }$ and $C^{\prime \prime }$ that are located within a user-defined maximum distance from an anchor, if they satisfy the following conditions:

1. The relative genomic order of the elements in each cluster is the same.
2. There are no elements belonging to another cluster between the the elements of $C^{\prime }$ and $C^{\prime \prime }$.
3. The total extension of the merge cluster $C^{\prime }\cup C^{\prime \prime }$ does not exceed a user-defined threshold.

#### 2.4.1. Counting Events Using Relaxed Adjacency Conditions

A less strict way of joining clusters is to require adjacency conditions of genomic anchors by only considering species that are involved in the clusters to join. Hence, we make sure that the clusters to join are joinable in all species that have an element in any of the considered clusters. In this way, we keep the syntenic orthology relation for the clusters and ignore species that do not appear in the relation. This leads to small changes of the estimated numbers of events, primarily as a result of the reduction of the number of singleton loci. On average, therefore, the numbers reported for duplications and insertions increases.

#### 2.4.2. Orthologs

**Figure 3:**
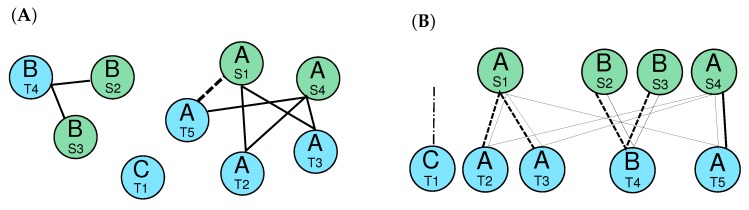
Example of the graph *G* for a cluster consisting of two groups of orthlogous elements in two species *S* and *T* (**A**). Thick edges indicate above-threshold sequence similarity. The dashed edge, which was included initially, must be inserted to correct *G*; otherwise T5-S4-T3-S1 would form a $P_{4}$. Modified Needleman–Wunsch alignment for graph *G* (**B**). The inserted edge to correct for a co-graph is now part of the thick edges showing the orthology relation. The alignment removes crossing edges of the orthology graph and detects duplications (dashed edges). The edge attached to node $T_{1}$ indicates a deletion in species *S* as there is no target node for this edge.

The resulting partition still may contain non-orthologous elements. In the case of tRNAs, for instance, the annotation generated by tRNAscan-SE only distinguishes anti-codon classes. These still may comprise multiple, discernible families. We therefore construct, for each cluster, a graph G=(V,E) whose vertices are the annotated elements that belong to the cluster. An edge is drawn between two elements *v* and *w* if their sequences are more similar than a certain threshold. In the case of tRNAs, values of 80% to 90% sequence identity have proved useful [23]. This value needs to be set as specifically dependent on the typical sequence conservation of the elements under consideration and on the phylogenetic range of interest. The graph *G* represents the orthology relation within a given cluster (see Figure 3A for an example).

As shown in [55], the graph *G* should be a co-graph; that is, it must not include a path $P_{4}$ on four vertices as an induced subgraph. If *G* is constructed from the sequence data using fixed thresholds for sequence similarity, it will sometimes violate the co-graph property. Nevertheless, it provides a good approximation. The initial graph *G* can be corrected by inserting or deleting the minimal number of edges that is required to restore the co-graph property. Although co-graph editing is known to be a difficult problem (the corresponding decision problem is non-deterministic polynomial-time hard (NP–hard) [56]), it remains tractable for sizes of candidate graphs that we typically encounter.

The possibly edited graph $G^{\prime }$ may still overpredict orthology in cases for which a cluster contains multiple types of elements that are distinguished by similarity. In such cases, the order relative to dissimilar elements may subdivide the ortholog clusters of $G^{\prime }$. To utilize this order information, we consider an alignment of the element that (i) preserves their genomic order, and (ii) allows matches only between elements that are connected by edges in $G^{\prime }$. This variation of the alignment problem is solved by a variation on the well-known Needleman–Wunsch alignment algorithm [57] that also allows duplications of elements (see Figure 3B for an example). As explained in Figure 3, the modified Needleman–Wunsch algorithm removes crossing edges and allows duplications. The exclusion of crossing is an intrinsic property of alignments and is the reason for choosing this type of approach here. More precisely, alignment algorithms compute maximum weight matchings that preserve the prescribed order in both sets, when presented with two linearly ordered sets of objects and a weighted bipartite graph of allowed matches of pairs of objects from different sets. The modified version of the Needleman–Wunsch algorithm employed here extends the match case in such as way that an element in one set may also be matched with one or more consecutive objects in the other set. We refer to [23] for the details on the dynamic programming solution to this problem.

### 2.5. Quantitative Analysis of Evolutionary Events

Taken together, the construction of the orthology relation outlined above provides, for each final orthology graph, information on (i) the first appearance of the ortholog group, (ii) duplication events, and thus (iii) the losses. This follows from the theory developed in [55,58] establishing the correspondence between orthology relations and event-labeled gene trees. Usually, one is primarily interested in placing duplication and loss events relative to a known gene phylogeny. Although it is not always possible to reconcile event-labeled gene trees with species trees [59], we found that our data were almost always “clean” enough to cause few problems in this respect, because the final ortholog groups contained only very small numbers of locally occurring paralogs. We could therefore use a simple heuristic that corrected the graph structure by deleting or adding edges in such a way that they could be reconciled into a phylogeny. The heuristic iteratively deletes or adds edges in order to edit the structure. At the same time, the number of edges to be edited is kept minimal.

Given a species tree *S* and cluster *C* of orthologous genes, we let σ(x)∈S be the species in which element x∈C resides. Thus σ(C) is the set of species in which members of the cluster are attested. The appearance or *insertion* of *C* into *S* occurs within the edge ancestral to the least common ancestor *ℓ* of σ(C) in *S*. As a consequence, every cluster that is present ancestrally is viewed as an “insertion before the root”. Using the same parsimony assumption, we assume that deletions of *C* appear in the edge ancestral to maximal subtrees $S^{\prime }$ of *S* below *ℓ* that do not contain species from σ(C). If the species tree is fully resolved, then deletions are never inferred at an edge leading to a child of *ℓ*.

**Figure 4:**
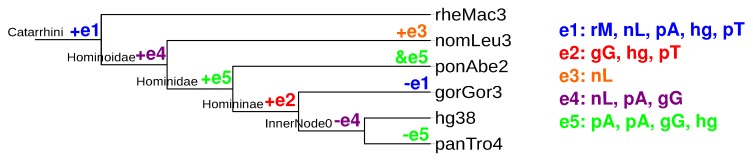
Example for counting genetic events: (e1)–(e5) are five groups of orthologous elements; ‘+’ and ‘-’ signs show where insertions and deletions are counted in the tree on the basis of the groups; ‘&’ depicts a duplication. Deletions can possibly also be reported as missing data; rM, nL, pA, gG, hg and pT are abbreviations for species identifiers rheMac3, nomLeu3, ponAbe2, gorGor3, hg38 and panTro4.

If a cluster contains multiple paralogs, duplication events are associated with changes in the copy number. Because clusters are by construction local in the genome, such duplication events correspond to tandem duplications. In contrast, the proliferation of the elements by insertion at different loci is accounted for by the insertion events. A detailed mapping of tandem duplications to the species trees is non-trivial, as the event-labeled gene trees obtained from co-graphs are usually not fully resolved. The pipeline therefore counts only the duplication events that occurred along the lineage leading from the root to a given leaf. This information can be extracted directly from the pairwise alignment of the element orders within each cluster. An example is shown in Figure 4.

### 2.6. Pseudogenes and Remolding Events

An important pathway to gene loss is pseudogenization, which can in many cases be detected by means of sequence similarity. Pseudogenes are identified on the basis of their sequence similarity to the target elements. If Infernal is used to retrieve a set of target elements, the user can specify a threshold for the Infernal score that will mark an element as a pseudogene. If target elements are given as a table created by the user, the table will include a column specifying whether the element at a given locus is considered a pseudogene or not. In the case of tRNA detection, tRNAscan-SE is used to retrieve a set of target tRNAs.

Remolding refers to an evolutionary event that changes the type or subtype of a molecule. The best-known examples are changes of the anti-codons in tRNAs such that the tRNA then refers to a different amino acid [60,61]. Remolding events are determined on the basis of the similarity thresholds for detecting orthologous elements and annotated element types. Hence, given two tRNAs with distinct types but a similarity above the specified threshold, the pair of tRNAs is reported as a remolding event. Conversely, if two elements have the same type but their sequence similarity is below the given threshold, this will be reported. The types of elements can at least in part be retrieved from Infernal or tRNAscan-SE output, which can be used by the user to generate a customized list of target elements. In the case that no type is given, remolding events cannot be reported. By definition, no remolding events can be associated with singleton clusters.

### 2.7. Implementation

Both parts of the pipeline run fully automatized according to the given input and parameters. Hence, the second part is available in two different versions: a fast version with as few output files as possible, and a slower, verbose version that will print intermediary files such that the user can have a deeper and more detailed look into the data. This includes the formation of clusters and graphs created thereof as well as derived duplication alignments used for counting phylogenetic events.

The current version of the pipeline requires the following input data:

1. A MSA of the genomes under consideration is required to extract the synteny anchor points. Currently only Multiz format is supported.
2. The corresponding genomic sequences are required for the annotation of the loci of interest. The pipeline expects fasta format. Because there is no guarantee that genome-wide MSA represents the complete genome, both MSA and genomes must be provided.
3. Target elements can be specified either as user-supplied annotation files or as one or more covariance models for annotation with Infernal or tRNAscan-SE. The modular organization of the pipeline makes it straightforward to add, in future releases, further means of generating annotation information, such as hidden Markov models of proteins.
4. A phylogenetic tree of the species of interest is necessary as a background to which evolutionary events are mapped.

The first three data items are required for the construction of the orthology relation. The phylogenetic tree is required only for the second part of the pipeline.

There are several parameters that can be adjusted by the user. The most important is the similarity threshold for true orthology candidates. For the showcase examples reported here, we used the same threshold value of 80%. The threshold for low-scoring MAF blocks that are to be discarded from the analysis can also be determined by the user. In addition, the pipeline offers several command-line parameters to only run on subsections of the workflow and to omit some of the intermediate processing steps. For details, we refer to the user manual.

The pipeline produces both machine-readable text files containing details of the analysis and condensed representations. The pipeline can also store detailed information on intermediate results that may also be useful in particular as a starting point to explore alternative analysis strategies. The final results include (i) the main results file, a phylogenetic tree displaying the evolutionary events in newick format, as well as auxiliary files for the visualization of the tree and event information using iTOL [62]; (ii) a file listing all gene clusters retrieved from the input data; (iii) a list of all genetic events sorted by event and species; (iv) a list containing the numbers of genetic elements sorted by species and type; and (v) a list containing remolding events. We also used iTOL [62], an interactive online visualization tool, to generate the results tree. Optional intermediate files include (i) the edge-weighted graph of each initial cluster; (ii) a file for each of the clusters specifying which elements are contained in the cluster, including all available annotation information for each element; (iii) the element-wise alignments of each cluster; and (iv) information on the co-graph structure or deviations thereof.

### 2.8. Benchmarking with Artifical Data

In order to test the functionality and performance of the pipeline, we constructed artificial data sets comprising six species with artificial “genomes” that were initially linked by 10,000 genetic anchors; 100 simulated “genetic elements” subdivided into three distinct types were randomly placed between the anchors. We considered both a random placement of the element and the insertion of elements into homologous positions of all or of a subset of the species. In order to model tandem duplication, furthermore, a fraction of elements were added twice. In order to simulate noise in the genome-wide alignments, a fraction of the anchor blocks were deleted randomly. We considered perfect data as well as a loss of 20% and 40% of the anchor blocks. For each setting, we executed our pipeline and compared the reconstructed orthology assignments and gain/loss statistics to the known ground truth.

## 3. Results

### 3.1. Automatic Pipeline for Multicopy Elements

We have developed a fully automatized pipeline that implements an improved version of the conceptual workflow of [23] for the detailed quantitative analysis of genetic elements that are subject to concerted evolution. It uses synteny information provided by uniquely aligned sequences adjacent to the multicopy elements of interest as the key information to disentangle their evolutionary relationships. The mathematical properties of orthology relations and their equivalents to event-labeled gene trees guide the post-processing of the data. This makes it possible to obtain an accurate and very well resolved picture of the history of multicopy families. In the work of [23], the workflow was not implemented in a coherent piece of software but was left at a conceptual level, requiring each analysis step to be performed in isolation. Here, we describe a fully automatized and publicly available pipeline that not only greatly facilitates the analysis in practice, but also ensures a high degree of reproducibility. For convenience, the pipeline also includes options to automatically generate input annotation data using tRNAscan-SE and Infernal. By including checks for missing data and distinct levels of adjacency constraints, we furthermore improved the accuracy of counting genetic events along the phylogenetic tree. Finally, the output of our pipeline includes files to easily visualize the resulting phylogenetic tree using iTOL, thus facilitating the interpretation of the results.

The pipeline, which is written in Python and Perl, is available from https://github.com/AnneHoffmann/Smore. It requires Infernal and tRNAscan-SE if the user decides to use these tools for the genome annotation step. A user manual provides detailed usage instructions. We additionally include a small example in the repository giving instructions on how to apply the pipeline to data. Input data and output files for all subcommands applied on the small test set are available. The repository also provides the covariance models and the gene lists used in this contribution. As showcase examples, we investigated the evolution of several multicopy ncRNA families. First, we reanalyzed the evolution of tRNAs in two different mammalian data sets, which were comprised of 6 and 10 species. Then we considered the much less widely studied Y RNAs for mammals and nematodes.

### 3.2. Application to Artificial Data

**Figure 5:**
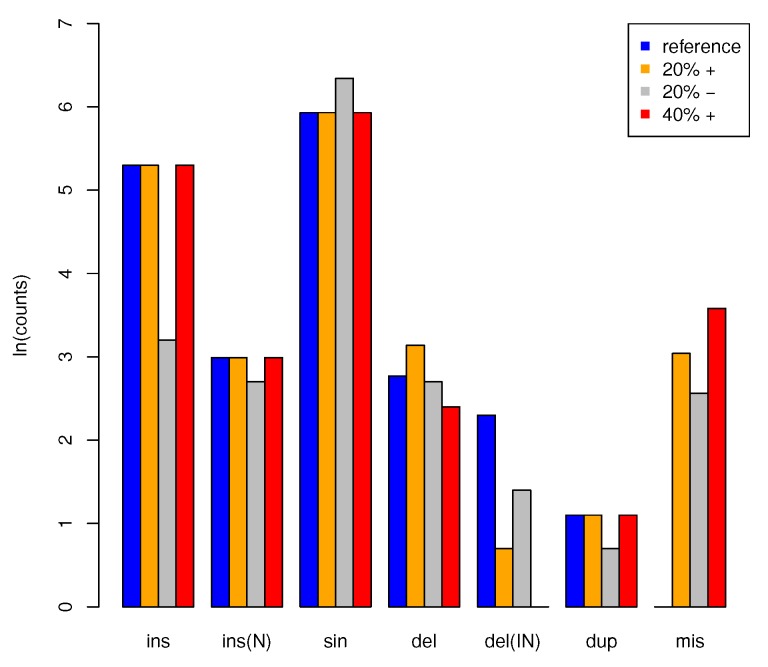
Summary of results for simulated data. The final counts (counted as natural logarithm ln(counts), *y*-axis) for evolutionary events, i.e., insertions (ins) and deletion (del) at the leaves, insertions (ins(IN)) and deletions (del(IN)) at the interior nodes, singletons (sin) and potentially missing data (mis) are compared between the reference ground truth and alignment with 20% (orange and grey) and 40% (red) of missing anchors. For 20% noise level we also compare the results with (+, orange) and without (−, grey) the segment joining step. High levels of noise mostly lead to a reduction in the inferred number of deletions and a corresponding increase in the reporting of missing data. Employing the joining strategy in general yields much more accurate results. Omitting the joining step in particular leads to fewer numbers of insertions inferred for interior nodes.

As described in Section 2.8, artificial data sets were created using distinct levels of noise, hence including perfect data, and a 20% and 40% loss of genomic anchors (see Figure 5). Using perfect data, that is, no deleted blocks, the pipeline exactly reconstructed the ortholog groups. With increasing noise level, the number of singletons decreased and the number of inferred local duplications increased, as loci were joined upon the loss of intervening anchors. With increasing noise level, an increasing fraction of deletion events were classified as missing data. At the same time, we observed an increase in inferred insertions at interior nodes of the tree, owing to a failure to correctly assign an ortholog from an outgroup. Both effects were expected and cannot be addressed at the level of synteny data. In order to counteract this issue, more accurate and complete genome-wide alignments would be necessary.

### 3.3. tRNAs

**Figure 6:**
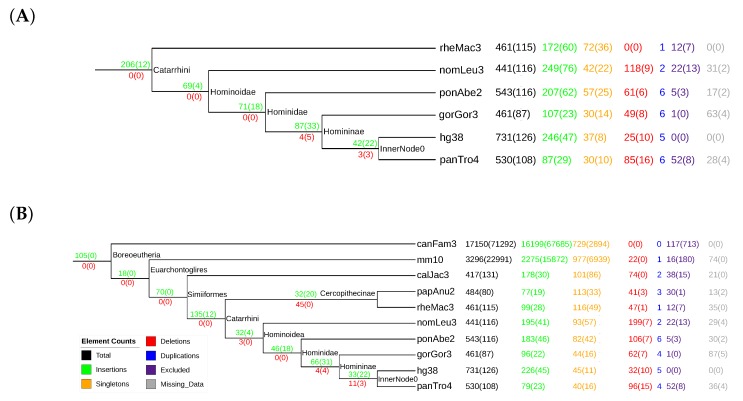
Summary of the evolutionary events inferred for transfer ribonucleic acids (tRNAs) in an evaluation with 6 (**A**) and 10 (**B**) species. Insertions and deletions that occur for groups of orthologous elements are inserted at their lowest common ancestor, and possible deletions are added below the interior branches to which they refer. Other events such as singletons and duplications are added directly at the leaves for each species separately. Orthology relations are based on a similarity threshold of 80% sequence similarity, and clusters were joined using the relaxed adjacency constraints. Numbers in parentheses are numbers of pseudogenes.

The comparison of the 6 and 10 species’ data shows an interesting effect: lineage-specific deletions of tRNAs seem to be very frequent in mammals (see Figure 6). Including three additional outgroups substantially increases the number of tRNA loci that predate the ancestor of the Catarrhini. While in the 6 species data set, 206 of the 731 human tRNAs are placed at the ancestral branch, the number increases to 328 in the 10 species set. This is compensated for by a correspondingly larger number of lineage-specific losses in the outgroup species and a reduction of predicted insertion events in the human lineage.

Remolding of tRNAs was analyzed for the 10 mammalian species’ data. Although the exact numbers depend on the choice of the similarity threshold and the details of the cluster-joining procedure, we recovered most of the remolding events previously described in [22,23]. Detailed data are provided in Supplementary Material S3. As in previous reports, the overwhelming majority of remolding events concern pseudogenes and/or are lineage-specific, and they most likely are the first steps in tRNA pseudogenization.

### 3.4. Mammalian Y RNAs

**Figure 7:**
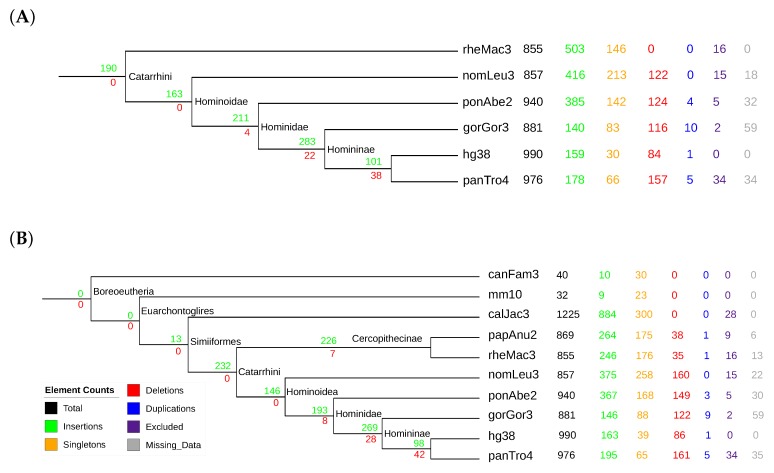
Summary of the evolutionary events inferred for Y ribonucleic acids (Y RNAs) in an evaluation with 6 (**A**) and 10 (**B**) species. See the caption of Figure 6 for a detailed legend. The main difference between the two data sets is that the inclusion of an additional member of the Cercopithecinae moves a substantial number of the insertion events from Hominoidae to Catarrhini.

Our data suggest that the spread of Y RNA sequences is an ongoing process in mammals. Of the 990 loci identified, 190 date back to the ancestor of the Catarrhini, while on the order of 100 loci have been inserted in both the human and the chimpanzee lineage after their divergence (see Figure 7). The 6 and 10 species’ data sets are largely consistent, although the inclusion of an additional member of the Cercopithecinae places many insertions that are estimated to be specific to Hominoidae to Catarrhini. Only a very moderate number of Y RNA loci were already populated in the ancestor of Simiiformes.

The copy numbers of the Y RNA families are comparable with the data reported in [63]. Within Catarrhini, there are consistently more Y1 and Y3 genes than Y4 loci. The number of Y5 copies remains small throughout the clade. Consistent with [63], our data show an appreciable level of synthenic conservation of Y RNA loci also beyond the Y RNA cluster that typically harbors a functional copy of each of the four families [44]. Complete data are provided in Supplementary Material S4.

### 3.5. Nematode stem-bulge RNAs

The stem-bulge RNAs (sbRNAs) were discovered in a systematic screening of a ncRNA-specific full-length complementary DNA (cDNA) library for *C. elegans* [64] and in a subsequent contribution [65] that listed additional experimentally verified members of this family. A detailed study of their sequence evolution [47] showed that their best-conserved sequence elements are similar to those of vertebrate Y RNAs, leading to the realization that they are in fact the homologs of the Y RNAs in other animal clades. Functional similarities are discussed in [66].

**Figure 8:**
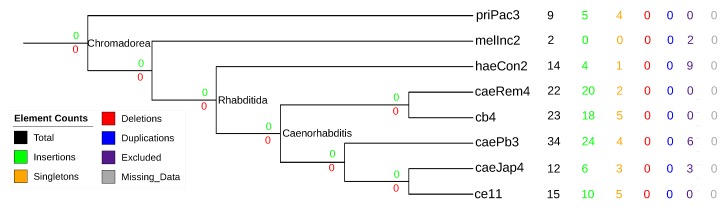
Evolutionary events of stem-bulge RNAs (sbRNA) in eight nematode species. See the caption of Figure 6 for a detailed legend.

Consistent with [47], we found arrays of tandem-duplicated sbRNAs in most species (see Figure 8). There are, however, no recognizable syntenically conserved orthologs. Given the large evolutionary distances and the high frequency of genome rearrangment [67], it is entirely plausible that this data set was too divergent to be informative for our method. This example draws attention to the limits of the synteny-based approach.

## 4. Discussion and Concluding Remarks

The methods of molecular phylogenetics require a strong correlation between sequence similarity and evolutionary divergence times. Because the mechanisms of concerted evolution obliterate this correlation, molecular phylogenetics is not applicable to the analysis of multicopy gene families including tRNAs and many other ancient ncRNA families. We have shown in a previous work that this limitation can be overcome in a systematic manner by using synteny, that is, conservation of relative gene orders, to identify orthologous elements [23]. In this contribution, we now report on the implementation of a computational pipeline that automatizes the corresponding workflow and thus makes synteny-based analysis of gene families available in practice at genome-wide scales. The Synteny Modulator Of Repetitive Elements (SMORE) pipeline, available from https://github.com/AnneHoffmann/Smore, is composed of two parts: The first component is concerned with the determination of orthology groups. The second component of SMORE implements methods for the identification of evolutionary events and their quantitative analysis. We demonstrated the functionality of the pipeline both on artificial data sets and using the analysis of tRNA and Y RNA genes as real-life showcase examples. The results are at least qualitatively consistent with previous studies and extend and refine these considerably.

The approach presented here assumes the perfect conservation of gene order in the vicinity of the elements of interest. While this is a very good approximation at smaller evolutionary scales, for example, among primate genomes, there are noticeable violations at larger scales, as exemplified by the example of nematode sbRNAs. At the same time, fewer synteny anchors are available for more distant genomes, because large fractions of the genome are diverged beyond the limits of reliable alignments. As a consequence, anchors are on average separated by larger genomic distances and thus are more likely to be separated by genome rearrangements. It may be possible to include explicit information on gene-order differences, as in a maximum likelihood for gene-order analysis (MLGO) [68] or similar approaches [69,70,71]. A second open problem concerns the exact mapping of the local duplication events to the species tree. On the one hand, the co-graph of a family does not necessarily provide full resolution [55]; on the other hand, the pairwise list alignments of the elements are not necessarily consistent. The reconciliation of pairwise alignments with duplication into a common multiple alignment with duplication is an as-yet-unresolved problem. An alternative approach is to use tools such as OrthoAlign [72], which also include genome rearrangements.

The SMORE pipeline sets the state for large-scale quantitative investigations into the evolution of multicopy gene families. In particular, it provides the data required to estimate gain and loss rates and the relative effects of, for example, unequal crossover (which governs local gain and loss), retroposition (leading to insertions at novel loci), and pseudogenization (leading to a loss of function and subsequent gradual disappearance of the element under consideration). This quantitative view is of particular importance for even larger families of repetitive elements.

## Supplementary Materials

The following are available online at www.mdpi.com/2075-1729/7/4/42/s1. S1: Stockholm alignment of sbRNA sequences; S2: Table of genome assemblies used; S3: Table of alloacceptor remoldings in detected tRNA genes; and S4: Distribution of different Y RNA types in mammals.
