# Supplementary material for: SMORE: Synteny Modulator of Repetitive Elements
Source: Life (Basel). 2017 Oct 31;7(4):42. doi: 10.3390/life7040042 (PMC5745555; doi:10.3390/life7040042)
Supplement: Supplementary file 1 [file life-07-00042-s001.zip › Supplement_review/S4.pdf]

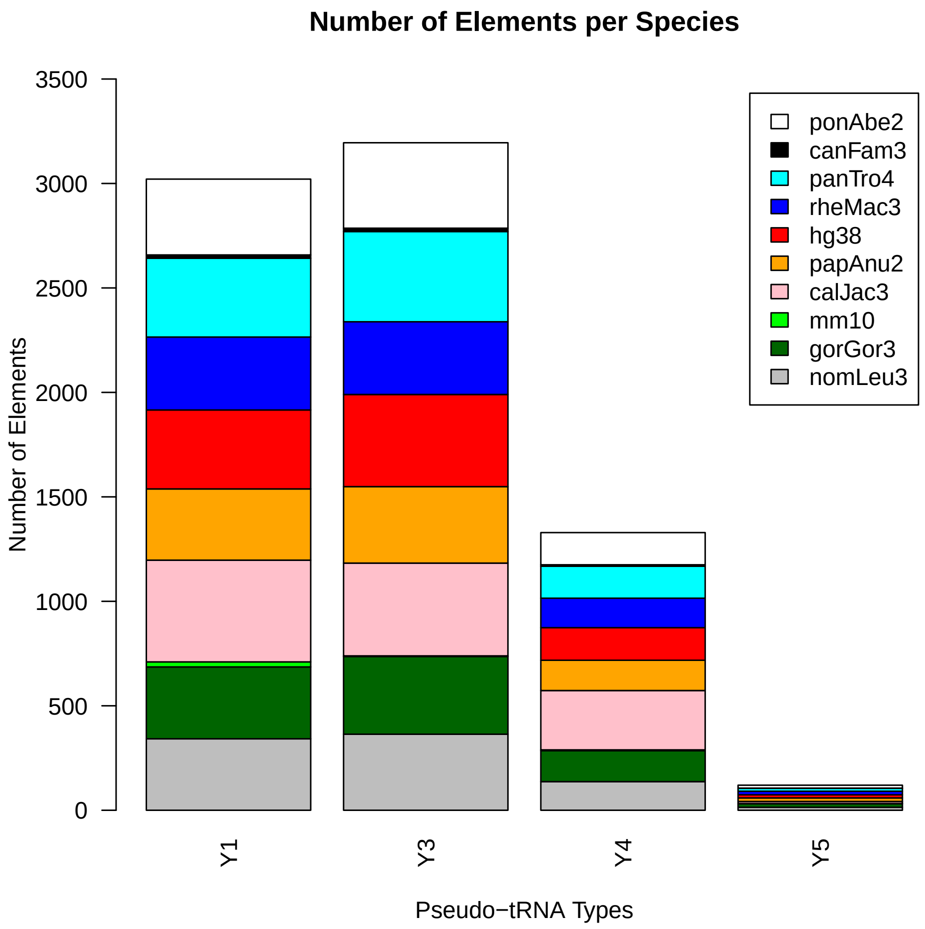

Distribution of different Y RNA types in ten mammalian species. The copy numbers of the Y1 and Y3 loci are some more than Y4. At least occur Y5 copies.
