# Supplementary material for: SMORE: Synteny Modulator of Repetitive Elements
Source: Life (Basel). 2017 Oct 31;7(4):42. doi: 10.3390/life7040042 (PMC5745555; doi:10.3390/life7040042)
Supplement: Supplementary file 1 [file life-07-00042-s001.zip › Supplement_review/S2.pdf]

The table showing the genome assemblies used in this analysis. The genome versions correspond to the `multiZ` alignments. The genome sequences were downloaded from the `UCSC genome browser`.

species	date	version
Caenorhabditis japonica	Aug. 2010	WUSTL 7.0.1/caeJap4
Caenorhabditis brenneri	Nov. 2010	C. brenneri 6.0.1b/caePb3
Caenorhabditis remanei	Jul. 2007	WS220/caeRem4
Callithrix jacchus	Mar. 2009	WUGSC 3.2/calJac3
Canis lupus familiaris	Sep. 2011	Broad CanFam3.1/canFam3
Caenorhabditis briggsae	Apr. 2011	WS225/cb4
Caenorhabditis elegans	Feb. 2013	WBcel235/ce11
Gorilla gorilla gorilla	May 2011	gorGor3.1gorGor3
Haemonchus contortus	Jul. 2013	WormBase WS239/haeCon2
Homo sapiens	Dec. 2013	GRCh38hg38
Macaca mulatta	Oct. 2010	BGI CR 1.0rheMac3
Meloidogyne incognita	Feb. 2008	M. incognita WS245/PRJEA28837/melInc2
Mus musculus	Dec. 2011	GRCm38/mm10
Nomascus leucogenys	Oct. 2012	GGSC Nleu3.0nomLeu3
Pan troglodytes	Feb. 2011	CSAC 2.1.4panTro4
Papio anubis	Mar. 2012	Baylor Panu_2.0/papAnu2
Pongo abelii	Jul. 2007	WUGSC 2.0.2ponAbe2
Pristionchus pacificus	Aug. 2014	P_ pacificus-v2/priPac3
