# Supplementary material for: SMORE: Synteny Modulator of Repetitive Elements
Source: Life (Basel). 2017 Oct 31;7(4):42. doi: 10.3390/life7040042 (PMC5745555; doi:10.3390/life7040042)
Supplement: Supplementary file 1 [file life-07-00042-s001.zip › Supplement_review/S3.pdf]

Alloacceptor remoldings in tRNA genes. The arrow indicates the direction of the remolding and the species in which the remolding was seen in comparison to other species in the tree. For entries without arrow, we only see two elements with similar sequence but different types, hence we cannot derive the derivation of the sequence. So types and species are both given.

Remolding	Species	[1]	[2]	Pseudogene
Leu → Phe	papAnu	-	-	F
Gly - Ala	ponAbe - canFam	+	-	F
Thr - Arg	ponAbe - gorGor	-	-	F
Ile → Ser	panTro	+	+	F
Asn - Ser	mm10	-	-	F
Phe - Val	papAnu - rheMac	+	-	F
Leu → Val	panTro	+	+	F
Cys → Tyr	ponAbe	+	+	F
Lys → Arg	rheMac	+	-	F
Met → Ile	gorGor	+	-	F
Thr → Ile	rheMac	+	-	F
His → Gln	rheMac	+	-	F
His → Arg	panTro	+	+	F
Ala → Val	mm10	+	+	T
Ala - Pro	canFam	-	-	T
Ala - Ser	mm10	-	-	T
Arg → Ile	mm10	-	-	T
Arg → Ser	mm10	-	-	T
Arg → Asn	mm10	-	-	T
Ser → Asn	mm10	-	-	T
Ser → Tyr	mm10	-	-	T
Ser → Lys	mm10	-	-	T
Ser → Thr	mm10	-	-	T
Ser → Gly	mm10	-	-	T
Cys → Tyr	hg38	+	+	T
Arg → His	canFam	-	-	T
Arg → Leu	canFam	-	-	T
Arg → Cys	canFam	-	+	T
Arg → Gly	canFam	+	+	T
His → Gln	canFam	+	-	T
Arg - Pro	canFam	-	-	T
Arg - Tyr	canFam	-	-	T
Gln - Trp	hg38 - gorGor	-	-	T
Val → Gly	panTro	+	+	T
Leu - Ser - Val	gorGor - panTro - hg38	+	+	T
Lys - Val	gorGor - hg38	-	-	T

References

[1] Velandia-Huerto, C.A., Berkemer, S.J., Hoffmann, A., Retzlaff, N., Romero Marroquín, L.C., Hernández Rosales, M., Stadler, P.F., Bermúdez-Santana, C.I.: Orthologs, turnover, and remolding of tRNAs in primates and fruit flies. BMC Genomics **17**, 617 (2016). doi:10.1186/s12864-016-2927-4

[2] Rogers, H.H., Griffiths-Jones, S.: tRNA anticodon shifts in eukaryotic genomes. RNA **20**, 269–281 (2014). doi:10.1261/rna.041681.113
